# Renal pyelocalyceal squamous cell carcinoma in a patient with an ectopic kidney presenting with chronic pyelonephritis: a case report

**DOI:** 10.1186/s13256-019-2090-z

**Published:** 2019-05-23

**Authors:** Yavuz Güler, Burak Üçpınar, Akif Erbin

**Affiliations:** 1 Department of Urology, Private Safa Hospital, Istanbul, Turkey; 2 Department of Urology, Haseki Training and Research Hospital, Istanbul, Turkey

**Keywords:** Ectopic kidney, Stone disease, Squamous cell carcinoma, Renal pelvic tumor

## Abstract

**Background:**

Until now, few cases of pelvis squamous cell carcinoma in various renal anomalies have been reported. To our knowledge, primary squamous cell carcinoma arising from a pelvic ectopic kidney has never been described. In this report, we describe a case of renal pyelocalyceal squamous cell carcinoma in a patient with an ectopic kidney presenting with chronic pyelonephritis.

**Case summary:**

A 73-year-old Caucasian woman presented to our hospital with pyelonephritis symptoms. Abdominopelvic computed tomography revealed heterogeneous and irregular minimal contrast enhancement in the pelvic ectopic kidney parenchyma. Radiologists reported that the images were consistent with chronic pyelonephritis. A Tc-99m dimercaptosuccinic acid renal scan demonstrated a nonfunctioning right pelvic ectopic kidney. The patient underwent open simple nephrectomy via modified Gibson incision. The whole mass was a distended, saclike structure without any grossly visible renal tissue. Pathological examination showed renal pelvis squamous cell carcinoma 8 cm in diameter infiltrating into the renal capsule and perinephritic fatty tissue. The patient was staged as T4N0M1 renal pelvis squamous cell carcinoma. The patient was being treated in the intensive care unit for respiratory distress on the seventh day after the operation. By the first-month follow-up visit, the patient had died of acute respiratory distress syndrome.

**Conclusions:**

Although rare, renal pelvis squamous cell carcinoma should be considered in the differential diagnosis of a renal mass in patients who have renal anomalies and chronic pyelonephritis.

## Introduction

Primary malignant tumors of the renal pelvis are relatively rare and constitute approximately 8–14% of all the renal malignancies [1]. Of these, renal pelvic squamous cell carcinomas (SCCs) are very rare tumors, accounting for 0.7–7% of cases among renal pelvic tumors in the general population. SCC usually occurs in late adulthood (aged 50–70 years).

Infectious etiologies such as tuberculosis, chronic pyelonephritis, and pyonephrosis have been linked to SCC, and a history of analgesic abuse with phenacetin or surgery for urolithiasis has been implicated in its development [2]. The clinical presentation is similar to that of urothelial carcinoma, and symptoms often include hematuria and abdominal or flank pain. A solid mass, hydronephrosis, and calcifications are common radiological findings, but they are nonspecific, which may explain why the diagnosis frequently is not established before histopathological examination of the surgical specimen. Early metastatic spread is common, and the prognosis is poor, with few patients surviving longer than 5 years. Until now, SCC cases have been reported in the kidneys with various anomalies such as horseshoe kidney, polycystic kidney disease, and renal calyceal diverticulum [3–5]. To our knowledge, this is the first reported case of SCC in a pelvic ectopic kidney. Along with a review of current literature on the topic, we report a case of SCC diagnosed after simple nephrectomy in a patient presenting with chronic pyelonephritis and nonfunctional kidney who had pelvic kidney.

## Case presentation

A 73-year-old Caucasian woman presented with a 3-month history of pain and fullness in the right lower quadrant of the abdomen, which had increased in recent days. The patient’s previous history included intermittent fever, tremor attacks, and use of multiple antibiotics. She was married and had two children (40 and 51 years old), both healthy. She did not smoke tobacco and consumed no alcohol. She had no history of surgery. Her father was 95 years old, and her mother had died at age 80 years of coronary artery disease. None of them had malignancies in their past history.

The patient appeared toxic; her temperature, pulse rate, respiratory rate, and blood pressure were 38.8 °C, 110 beats/min, 30 breaths/min, and 90/50 mmHg, respectively. A physical examination revealed that the patient had a palpable mass in the right lower abdominal quadrant.

**Fig. 1:**
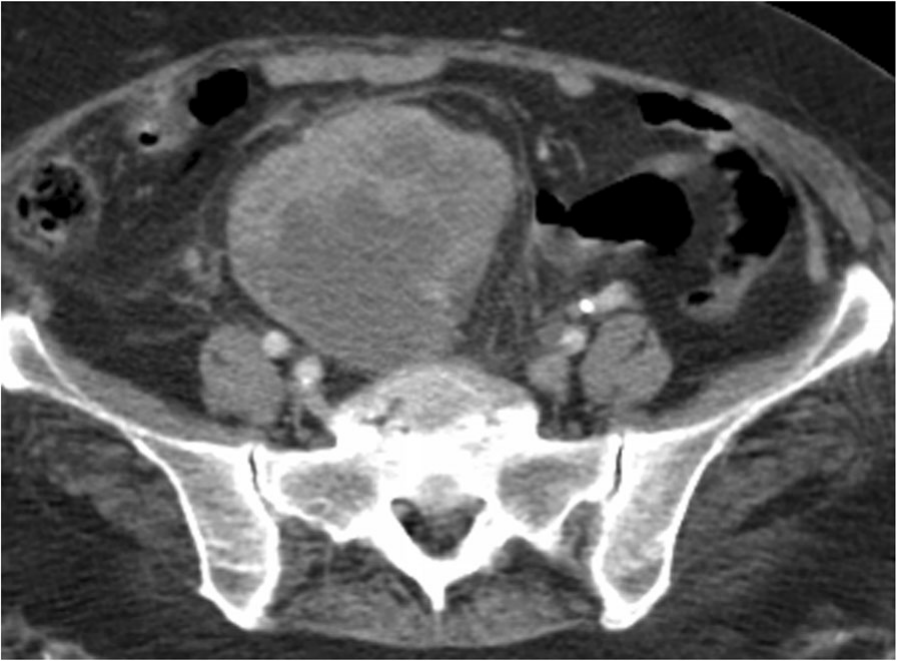
Contrast-enhanced computed tomography. There are hypodense areas in the pelvic kidney parenchyma with irregular limited contrast enhancement. The renal parenchyma shows heterogeneous and minimal contrast enhancement. There is no obvious mass lesion. The findings were consistent with chronic pyelonephritis

The initial laboratory test results showed significant leukocytosis with a white blood cell (WBC) count of 37,100/μl, elevated C-reactive protein (CRP) of 218 mg/L, and mildly elevated creatinine of 1.2 mg/dl. Abdominopelvic computed tomography (CT) revealed heterogeneous and irregular minimal contrast enhancement in the pelvic ectopic kidney parenchyma. Radiologists reported that the images were consistent with chronic pyelonephritis (Fig. 1). A Tc-99m dimercaptosuccinic acid (DMSA) renal scan demonstrated a nonfunctioning right pelvic ectopic kidney.

The patient underwent open simple nephrectomy via modified Gibson incision.

**Fig. 2:**
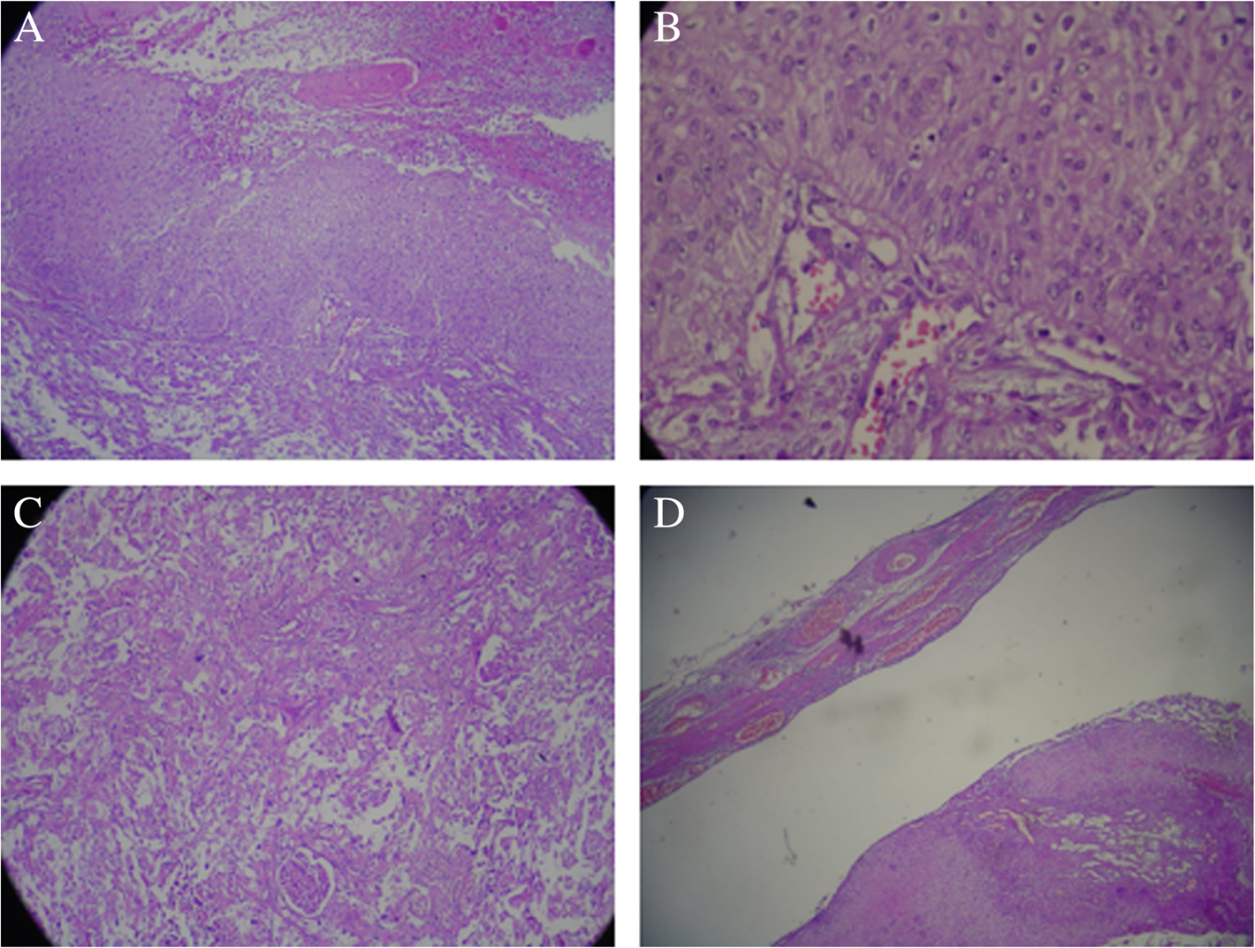
Microscopic findings of resected renal pelvic tumor (H&E stain). Squamous cell carcinoma is chosen in well-differentiated areas of the tumor (**a**, 10 × 10 μm; H&E stain; **b**, 40 × 10; H&E stain). Sarcomatoid morphology and residual glomerular structures are seen in poorly differentiated areas (**c**, 10 × 10 μm; H&E stain). Squamous differentiation in tumoral areas is prominent (**d**, 10 × 10 μm; H&E stain)

During surgery, a huge kidney specimen measuring 15 × 10 × 8 cm was obtained. The whole mass was a distended, saclike structure without any grossly visible renal tissue. Pathological examination showed renal pelvis SCC 8 cm in diameter infiltrating the renal capsule, in addition to perinephritic fatty tissue. Microscopic examination revealed SCC structures in well-differentiated areas and sarcomatoid changes in poorly differentiated areas. The nuclear grade of the tumor was 4, and it had focal sarcomatoid morphology (Fig. 2). However, other microscopic findings supported the presence of concurrent chronic pyelonephritis. ^18^F-fluorodeoxyglucose (FDG) positron emission tomography/CT showed a hypermetabolic mass measuring 4 × 3 cm in the right parahilar area of the thorax, hypermetabolic nodules in both lung parenchyma, and increased FDG uptake in iliac, acetabulum, femur, head, neck, sternum, and costal bones. The patient’s diagnosis was accepted as T4N0M1 renal pelvis SCC, and she was admitted to the intensive care unit for respiratory distress on the seventh postoperative day. She had died of acute respiratory distress syndrome by the first-month follow-up visit. No autopsy was performed after the patient died.

## Discussion

Until now, few cases of pelvic SCC in various renal anomalies have been reported.

Twenty-four cases of renal pelvic tumor in horseshoe kidneys have been reported previously in a Japanese series. Thirty percent of the cases included components of SCC. The authors concluded that the high incidence of SCC might be related to the high incidence of stone formation or chronic irritability in patients with horseshoe kidney [3]. Chen *et al.* reported a case of primary SCC arising from a calyceal diverticulum in a patient whose initial imaging showed a benign-appearing cystic lesion [4]. Xie *et al*. reported a case of renal pelvis SCC with tumor embolus in a 35-year-old patient with autosomal dominant polycystic kidney disease [5].

Our patient was a 73-year-old Caucasian woman with a right ectopic huge kidney. She presented with septic symptoms such as fever and fatigue. When admitted to the hospital, her CRP and WBC values were 218 mg/L and 37,100/μl, respectively. Initial radiologic examinations such as contrast-enhanced CT and urinary ultrasonography revealed that this disease was not cancer. In the subsequent DMSA renal scintigraphy, we found that the kidney was not functioning, and therefore we decided to perform a simple nephrectomy. The patient underwent open simple nephrectomy, and the pathology was found to be renal pyelocalyceal SCC. To the best of our knowledge, primary SCC arising from a pelvic ectopic kidney has never been described until now. Thus, to the best of our knowledge, the presented case is the first such report in the literature.

SCCs of the urinary tract is commonly observed in the bladder and male urethra and is rarely observed in the renal pelvis. Chronic renal stone disease, phenacetin use, radiotherapy, immunosuppressive treatment, infections, vitamin A deficiency, exogenous chemicals, hormonal imbalance, and rarely schistosomiasis are risk factors for SCC [6]. In our patient, recurrent urinary tract infections were the only underlying potential cause. Chronic irritation, inflammation, and infection of the urothelium cause squamous metaplasia, and this pathology progresses to dysplasia and eventually evolves to squamous carcinoma. Patients typically present with flank pain, fever, chills, and hematuria. In advanced cases, patients may be admitted with flank mass, malaise, anorexia, and cachexia. Additionally, paraneoplastic findings such as hypercalcemia, leukocytosis, and thrombocytosis may be observed [7]. SCC of the kidney can originate from a primary lung SCC as well.

However, in our patient, previous thoracic imaging did not reveal any pathologies in the lung parenchyma. Therefore, we hypothesized that the primary origin of the tumor was the kidney. Other sites of metastasis originated from this primary tumor by vascular spread.

An exact diagnosis of SCC cannot be made by preoperative imaging methods or clinical findings. As in our patient, diagnosis is commonly achieved by histopathologic examination of the nephrectomy specimen, which is commonly applied for a nonfunctioning kidney.

Radiologic findings of SCC can range from solid mass with calcifications and concomitant hydronephrosis to infiltrative mass with indistinct borders, parenchymal mass with perirenal invasion, and nonfunctioning kidney with renal stones. On gross pathologic examination, SCC is commonly observed as a necrotic, ulcerative mass that invades the renal pelvis.

Diagnosis of renal pelvis SCC is often delayed until the tumor invades the renal parenchyma or surrounding tissues. Owing to its aggressive behavior, SCC may spread to regional lymph nodes, lungs, and the liver, and rarely to bones [7]. Five-year overall survival is approximately 10%, and most of these patients die within 1 year of diagnosis [8]. Even when caught in its early stages (T1–T2), patients may not benefit from curative treatment, which is nephrectomy.

Therefore, aggressive surgical resection is required in patients who are scheduled for surgery. Radiotherapy and chemotherapy also have very limited use in SCC treatment. As chemotherapy, cisplatin-based regimes (for example, cisplatin-5fu, MVAC) can be used, but outcomes are not satisfactory. Patients with asymptomatic kidney stones and chronic urinary tract infections should be closely followed. Raghavendran *et al.* have stated that patients with long-term kidney stones with low-functioning kidneys and associated hematuria should be evaluated by routine CT to determine possible malignancies [9].

## Conclusions

Renal pelvic SCC is a rare entity with an aggressive behavior. Nonspecific radiologic findings lead to late detection, often when the tumor becomes locally advanced or metastatic. Renal pelvis SCC should be considered in the differential diagnosis of renal masses in patients who have renal anomalies and chronic pyelonephritis. The prognosis of SCC of the renal pelvis is very poor. Aggressive surgical resection is the mainstay of therapy in renal pelvis SCC and may result in cure in low-stage patients. Adjuvant chemotherapy or radiotherapy has no concrete evidence of survival benefits; however, it may still play a role in the control of local symptoms in metastatic patients.

## Abbreviations

CRP: C-reactive protein
CT: Computed tomography
DMSA: Tc-99m dimercaptosuccinic acid renal scan
FDG: ^18^F-fluorodeoxyglucose
SCC: Squamous cell carcinoma
WBC: White blood cell

## References

[1] Tyagi N, Sharma S, Tyagi SP, Maheshwari V, Nath P, Asharf SM, *et al.* A histomorphologic and ultrastructural study of the malignant tumors of the renal pelvis. J Postgrad Med. 1993;39:197–201. 7996496

[2] Holmäng S, Lele SM, Johansson SL. Squamous cell carcinoma of the renal pelvis and ureter: incidence, symptoms, treatment and outcome. J Urol. 2007;178:51. 10.1016/j.juro.2007.03.033 17574059

[3] Mizusawa H, Komiyama I, Ueno Y, Maejima T, Kato H. Squamous cell carcinoma in the renal pelvis of a horseshoe kidney. Int J Urol. 2004;11:782–4. 10.1111/j.1442-2042.2004.00873.x 15379945

[4] Chen YW, Shen SH, Chang YH, Pan CC. Squamous cell carcinoma arising from a renal calyceal diverticulum. Urology. 2016;95:e5–6. 10.1016/j.urology.2016.05.055 27289028

[5] Xie J, Zhang XB, Wang WZ, Li HZ. Case report of renal pelvis squamous cell carcinoma with tumor embolus in autosomal dominant polycystic kidney disease. Medicine (Baltimore). 2016;95(33):e4597. 10.1097/MD.0000000000004597 PMC5370818 27537592

[6] Kanodia KV, Vanikar AV, Patel RD, Nigam LK, Trivedi HL. Rare co-existence of squamous cell carcinoma with infiltration of renal vein and xanthogranulomatous pyelonephritis. J Clin Diagn Res. 2015;9:ED15–6. 10.7860/JCDR/2015/14908.6924 PMC4717793 26816904

[7] Hameed ZB, Pillai SB, Hegde P, Talengala BS. Squamous cell carcinoma of the renal pelvis presenting as sacral bone metastasis. BMJ Case Rep. 2014;2014:bcr2013201719. 10.1136/bcr-2013-201719 PMC3918639 24493112

[8] Mardi K, Kaushal V, Sharma V. Rare coexistence of keratinizing squamous cell carcinoma with xanthogranulomatous pyelonephritis in the same kidney: report of two cases. J Cancer Res Ther. 2010;6:339–41. 10.4103/0973-1482.73351 21119270

[9] Raghavendran M, Rastogi A, Dubey D, Chaudhary H, Kumar A, Srivastava A, *et al.* Stones associated renal pelvic malignancies. Indian J Cancer. 2003;40:108–12. 14716114

